# Treatment of Anxiety in Athletes Using Cognitive Behavioral Therapy: A Single-Case Experimental Design with Multiple Baselines

**DOI:** 10.17505/jpor.2026.29449

**Published:** 2026-08-24

**Authors:** Rebecka Ekelund, Stefan Holmström, Andreas Stenling

**Affiliations:** 1 Department of Psychology, Umeå University, Umeå, Sweden; 2 Umeå School of Sport Sciences, Umeå University, Umeå, Sweden; 3 Department of Sport Science and Physical Education, University of Agder, Kristiansand, Norway

**Keywords:** Psychotherapy, N-of-1, ecological momentary assessment, experience sampling method, mental health, sport

## Abstract

Anxiety is one of the most prevalent mental health problems in athletes, yet the inclusion of athletes with clinical mental health problems in intervention studies remains limited. Furthermore, no studies to date have investigated the mechanisms of cognitive behavioral therapy (CBT) on anxiety and positive affect in athletes. Hence, the aim of the current study was to examine the effect of CBT on moderate to severe symptoms of anxiety and positive affect in athletes, as well as to investigate the mediated effects of CBT on anxiety symptoms and positive affect through rumination and acceptance. A randomized single-case multiple-baseline design was conducted with six athletes who experienced moderate to severe anxiety. Data was collected four times daily through ecological momentary assessment, and all participants received six weekly sessions of CBT. Intervention effects were analyzed using Tau and baseline-corrected Tau, and mediated effects were analyzed using a two-step approach with Tau and cross-lagged correlations. An intervention effect on anxiety was observed for two participants, while two participants showed an increase in anxiety. An intervention effect on positive affect was observed for three participants. In terms of mediation, only one participant showed evidence consistent with a mediated process in the expected direction through rumination on anxiety. These findings highlight individual variability in treatment effects and the underlying mechanisms that may help explain how interventions exert their effects. The current study underscores the complementary role of idiographic approaches alongside groupbased designs in advancing evidence on individual treatment effects and underlying mechanisms.

## Introduction

Anxiety disorders and symptomatic anxiety are among the most prevalent mental health problems in athletes, with prevalence rates comparable to those observed in the general population (Gouttebarge et al., 2019; Reardon et al., 2024; Rice et al., 2019). Yet, athletes remain underrepresented in clinical research evaluating cognitive behavioral therapy (CBT) for anxiety, limiting knowledge about treatment effects within the context of competitive sport (Ekelund et al., 2025a). More specifically, reported prevalence rates in the athlete population range from 6.6% (Oevreboe et al., 2023) to 9% when clinically assessed (Schaal et al., 2011), and up to 16.9% when self-reported (Åkesdotter et al., 2020). In a Swedish context, 69% of elite athletes who sought treatment at a psychiatric outpatient clinic specializing in elite sport during a six-year period (2015 to 2021) were diagnosed with an anxiety disorder according to criteria of the International Statistical Classification of Diseases and Health Related Problems 10th revision (ICD-10; e.g., social anxiety disorder, generalized anxiety disorder, panic disorder; Åkesdotter et al., 2022).

Given the attention anxiety symptoms and disorders have received within the context of competitive sport and athletes’ mental health (e.g., Reardon et al., 2024), it is surprising that clinical intervention studies including athletes with subclinical and clinical mental health problems are lacking (Ekelund et al., 2025a; Rice et al., 2019). A recent review investigating interventions for improving athletes’ mental health identified only three studies using clinical inclusion criteria, all focused on eating disorders, and only two studies that investigated mediators (Ekelund et al., 2025a). Thus, there are critical knowledge gaps in the literature regarding the efficacy of CBT and its effective mediators for a subclinical and clinical population of athletes. To address these knowledge gaps, the current study focuses on athletes with moderate to severe levels of anxiety and investigates the effects of CBT on anxiety symptoms and positive affect, as well as potential mediators of the treatment effects.

One explanation for the lack of inclusion of subclinical and clinical samples of athletes in intervention studies can be that the number of athletes eligible is often too small to yield scientific credibility in traditional group-based designs (e.g., randomized controlled trials; Currie et al., 2021). Single-case experimental designs (SCED; Kazdin, 2021a) offer a rigorous alternative for this population and are particularly well suited to contexts characterized by limited existing evidence, such as elite sport environments, or settings that differ from those typically represented in traditional group-based clinical research (Mirza et al., 2017). Moreover, SCEDs emphasizes the individual in their unique context (Kazdin, 2021a; Vlayen et al., 2020). For athletes specifically, they operate in a high-pressure, “win-at-all-costs” environment (Douglas & Carless, 2006), in which behaviors that may be considered functional or dysfunctional can differ from how such behaviors are viewed outside of the sport context (e.g., rituals, sacrifice, dedication; Carless & Douglas, 2013; Lebrun & Collins, 2017). More specifically, the context of competitive sport is characterized by rigorous training and competition schedules, risk of injury, rehabilitation demands, media scrutiny, fan expectations, and uncertainty surrounding funding and athletic careers (Henriksen et al., 2026; Schinke et al., 2024). Although context-specific demands and stressors can be found across many life domains (e.g., Sheldon et al., 2021; Syed et al., 2020), the stressors described above are unique to competitive sport and the athlete population. Exposure to any one of these factors, or their combination, can understandably place athletes under considerable psychological strain.

Because athletes’ psychological states and behaviors are strongly influenced by the contexts in which they operate (e.g., Carless & Douglass, 2013; Forsberg et al., 2025; Küettel & Larsen, 2020), the context of competitive sport provides an unexplored and novel context in which psychotherapy can be investigated. While empirical evidence about psychotherapy in the context of competitive sport is scarce, athletes’ lived experiences of psychotherapy provide important insights into how psychological treatment is perceived and experienced in practice by athletes. When interviewed about their experiences, five Swedish elite athletes reported negative experiences with general healthcare providers, primarily due to a perceived lack of understanding of elite sports which in turn influenced their perceived effectiveness of psychotherapy (Lundqvist et al., 2024b). These findings underscore the importance of clinicians understanding both clinical mental health and sport-specific demands for athletes to feel understood in the therapeutic environment (Cosh et al., 2024; Henriksen et al., 2026; Lundqvist et al., 2024b) and point to the need to examine the effectiveness of psychotherapy in this context (Ekelund et al., 2022).

The experiences of psychotherapy described by athletes combined with context-specific factors add to the discussion whether psychotherapy for athletes should be adapted to sport-specific demands or grounded in established psychotherapeutic interventions (Ekelund et al., 2022; Lundqvist & Meyer, 2025; Lundqvist et al., 2024a). Several arguments have been raised for adapting psychotherapy to athletes, including calls for disorder-specific treatments (e.g., Rice et al., 2016) as well as practical challenges related to athletes’ demanding schedules, frequent travel, and competition commitments, which may complicate access to regular therapy (Wang et al., 2024). However, given the extensive evidence base for established psychotherapies such as CBT (Fordham et al., 2021), clinical sport psychology would benefit from systematically evaluating existing psychotherapies in the context of competitive sport prior to developing new sportspecific treatment models (Ekelund et al., 2022; Lundqvist & Meyer, 2025).

However, while athletes may share contextual similarities, they remain a highly heterogeneous group. The psychological and performance-related demands athletes face can vary significantly depending on the level of competition (Swann et al., 2015), sport-specific cultural norms, and structural differences across sports and regions (Lundqvist et al., 2024). This underscores the importance of approaching athletes not merely as performers within a system, but as individuals with distinct psychological functioning and lived experiences. Hence, assuming the need for specific adaptations in athlete psychotherapy without empirical evidence lacks evidence-based justification (Ekelund et al., 2022). A more valid approach is to target the underlying change processes that drive individual functioning within each athlete’s unique context. In doing so, the evidence-based psychotherapeutic care for athletes who experience subclinical and clinical mental health problems will be strengthened.

### CBT and psychological change processes

CBT has become an umbrella term that includes the more traditional cognitive and behavioral therapies (Hayes & Hofmann, 2021) but also the so-called “third-wave” therapies (e.g., acceptance and commitment therapy [ACT], Hayes et al., 1999). For the remainder of this paper, we will use the term CBT when referring to both the traditional- and third-wave therapies (Hayes & Hofmann, 2021). In some instances where distinction is needed for clarification (e.g., referring specifically to ACT), this will be done.

The effectiveness of interventions such as CBT is well established across a variety of mental health problems and psychiatric disorder (Gloster et al., 2020; Fordham et al., 2021), including anxiety disorder (Swain et al., 2013; Ruiz & Odrizola-Gonzalez, 2017), when compared to control groups (Fordham et al., 2021), essentially showing that treatment is better than no treatment (Cuijpers, 2025). However, these findings are typically based on averaged group-level data, which masks how individuals within treatment groups respond to the treatment. Hence, although the efficacy of CBT is well established at the group level, less is known about how and for whom treatment effects emerge at the individual level, or about the underlying change processes that explain therapeutic outcomes (Kazdin, 2009; Moskow et al., 2023).

Psychological change processes are defined as “a set of theory-based, dynamic, progressive, contextually bound, and multilevel changes that occur in predictable empirically established sequences oriented toward the desirable outcomes” (Hofmann et al., 2020, p. 5). Despite efforts to identify psychological change processes (e.g., behavioral activation, problem solving, self-compassion, psychological flexibility; Moreno-Peral et al., 2020) in psychological interventions, little is still known about the underlying change processes over time and how they help interventions exert their effect (Hayes et al., 2019; Johannsen et al., 2022).

However, rumination and acceptance are two psychological change processes proposed by Hayes & Hofmann (2021). Additionally, both rumination and acceptance can be viewed as emotion regulation strategies, which in turn are considered transdiagnostic processes (Cludius et al., 2020). More specifically, rumination is defined as “the process of thinking perseveratively about one’s feelings and problems rather than in terms of the specific content of thoughts” (Nolen-Hoeksema et al., 2008) and is an emotion regulation strategy commonly used with anxiety disorders (Boemo et al., 2022).

Acceptance on the other hand is “the willingness to experience affect without needles escape, avoidance or constraint” (Hayes & Hofmann, 2021, p. 370) and “involves an active and aware embrace of those private events” (Hayes et al., 2006). Acceptance is one of the putative processes in ACT (Hayes et al., 1999) and has shown mediating effects across different types of treatment outcomes (e.g., quality of life, well-being, symptom reduction, behavioral changes; Stockton et al., 2019).

To understand these change processes, they must be studied at the level where they occur, that is, at the individual level (Hamaker, 2025). Accordingly, recent literature has emphasized the importance of a more individualized, process-based approach to treatment in combination with idiographic and longitudinal methodologies (e.g., Fisher et al., 2019; Hofmann & Hayes, 2019; Levinson et al., 2025; Moskow et al., 2023; Ryum & Kazantzis, 2024). Furthermore, increased use of SCEDs to investigate effects of psychotherapy in athletes have been encouraged given the current lack of knowledge (Ekelund et al., 2022, 2025a, 2025b; Wood & Turner, 2025). Hence, this study directly responds to calls for further research of this nature by conducting a SCED in a real world-clinical setting with high-density data collection through ecological momentary assessment (EMA; Shiffman, 2008). In doing so, the study contributes to a more nuanced and individualized understanding of CBT, advancing both clinical and sport psychology through increased knowledge of the processes underlying therapeutic change.

### The current study

The current multiple baseline SCED has two aims. First, we aim to examine the effects of CBT on moderate to severe symptoms of anxiety and positive affect in athletes. Second, we aim to investigate mediated effects of CBT on anxiety symptoms and positive affect through rumination and acceptance. To achieve these aims, the study seeks to answer the following research questions:

(i) Does CBT reduce symptoms of moderate to severe anxiety and increase levels of positive affect in athletes?
(ii) Do rumination and acceptance mediate the effects of CBT on moderate to severe symptoms of anxiety and positive affect?

## Materials and Methods

A study protocol was developed based on the guidelines of Johnson and Cook (2019), with added sections (i.e., design, context, analyses) from Tate et al. (2016). The original protocol was approved by all authors and preregistered on the Open Science Framework (OSF) on February 24, 2025 (https://osf.io/nwqrp/overview?view_only=668945f1e8514d03bec0410db984962a). Edits to the study protocol were made up until December 4, 2025. All edits can be found in the document *Transparent* at https://osf.io/nwqrp/overview?view_only=668945f1e8514d03bec0410db984962a. Finally, we have adopted the Single-Case Reporting Guide-line In Behavioural Interventions (SCRIBE; Tate et al., 2016) in the reporting of this SCED.

### Study design

A single-case concurrent multiple-baseline across participants with a randomized staggering introduction of the intervention was conducted. Phase change was determined a priori based on randomized length of baseline. Minimum length of baseline was five days, and maximum length was 20 days. Minimum length of the study was 48 days, and the maximum length was 64 days. However, Participant 6, who had the longest baseline, had to reschedule one session due to illness. Consequently, the study was extended to a total of 70 days for this participant. Neither the outcome assessors (RE and AS), the participants, nor the intervention facilitator were blinded to the onset of the intervention due to the nature of psychotherapy. However, the intervention facilitator was blinded to outcomes and data collection.

### Randomization

Case-randomization (i.e., participants are randomly assigned to start the intervention first, second, third, and so on; Levin & Ferron, 2021) was used and randomization was adapted to fit the clinic and the participants’ schedules. Intervention start was only possible on a Monday or Tuesday due to logistical constraints at the clinic. The participants were assigned to either group A (start on a Monday) or group B (start on a Tuesday). Three participants had other commitments on Mondays and were therefore assigned to group B. The three remaining participants were assigned to group A. Participants within each group were then randomly assigned to one of three possible starting points (i.e., study day 6, 13, or 20 for group A and study day 7, 14, or 21 for group B). The length of baseline was a trade-off between maximising the number of starting points (i.e., six) and the routines of the clinic where sessions were only available on Mondays or Tuesdays.

### Selection criteria

Inclusion criteria were: (1) over 18 years of age, (2) active competitive athlete, (3) experiencing moderate to severe symptoms of anxiety (i.e., cut off score of 10 on the seven-item Generalized Anxiety Disorder 7-item-scale; GAD-7) and/or fulfill criteria of psychiatric diagnoses related to anxiety in M.I.N.I., and (4) being able to attend weekly physical sessions at the clinic.

Exclusion criteria were: (1) under the age of 18, (2) not being able to attend physical sessions at the clinic, (3) ongoing psychological or psychopharmaceutical treatment, (4) received psychotherapy in the past 12 months, (5) mild levels of anxiety symptoms (i.e., a score of <10 on GAD-7) or anxiety symptoms solely related to competition (i.e., performance anxiety), or (6) psychiatric diagnoses not related to the study’s aim (e.g., eating disorders, obsessive-compulsive disorder) and/or neuropsychiatric disorders (e.g., attentiondeficit hyperactivity disorder, autism spectrum disorder).

### Participant characteristics

Six female athletes experiencing moderate to severe anxiety took part in the study. The limited number of elite athletes at the highest-level in Sweden necessitates careful consideration to ensure participant anonymity. Hence, information that could reveal the athletes’ identity (i.e., type of sport) was omitted. Although participant P4 met diagnostic criteria for obsessive-compulsive disorder (OCD), which was specified as an exclusion criterion, the participant was included because OCD was not considered the primary diagnosis nor the main focus of treatment according to the clinical assessment. Participant characteristics are presented in Table 1.

**Table 1:** Characteristics of participants at baseline

Participants	Age	Gender	Sport	Competition level	GAD-7 score	M.I.N.I assessment
P1	19	Female	Team	International	10	MDD
P2	24	Female	Individual	International	10	MDD, agoraphobia, history of panic disorder
P3	32	Female	Individual	National	15	History of MDD and anorexia nervosa
P4	22	Female	Individual	National	10	OCD, GAD, history of MDD and panic disorder
P5	32	Female	Team	International	6	Panic disorder, PTSD, history of MDD
P6	22	Female	Individual	National	12	GAD, history of MDD

*Note*. GAD = generalized anxiety disorder, MDD = major depressive disorder, OCD = obsessive compulsive disorder, PTSD = post-traumatic stress disorder

### Context

The location of the study was a private practice clinic specializing in clinical sport psychology located in a large city in Sweden. A licensed clinical psychologist with seven years of experience in both clinical and sport psychology delivered the intervention. All sessions took place in a smaller therapy room where the participants met individually with the psychologist.

### Procedure

All procedures were approved by the Swedish Ethical Review Authority (dnr: 2022-01768-01) and all participants provided informed consent. Participants were recruited through advertisement with a study information poster on social media (i.e., Instagram and LinkedIn) in collaboration with a clinic specializing in sport psychology. The study poster specified that we were seeking competitive athletes who were experiencing anxiety and interested in undergoing treatment for their symptoms. The advertisement was posted through the clinic’s social media channels for 15 days in January and February of 2025 and was shared by the study authors on their LinkedIn profiles.

Participants initially contacted the first author by email. If any exclusion criteria were stated in the first contact (e.g., not able to attend physical sessions, not competitive athletes) the participants were informed that they could not participate in the study. Participants were invited to take part in an individual online clinical assessment conducted by the second author, who is a licensed clinical psychologist with many years of experience in clinical assessment and sport psychology. The clinical assessment began with the collection of descriptive information (i.e., age, gender, type of sport, level of competition). Thereafter, participants completed the seven-item Generalised Anxiety Disorder 7-item scale (GAD-7; Spitzer et al., 2006) followed by a structured diagnostic interview using the M.I.N.I Swedish translation, version 7.0.0 (Sheehan et al., 1998). Upon completing the clinical assessment, all participants who fulfilled inclusion criteria were asked to participate in the study.

**Figure 1:**
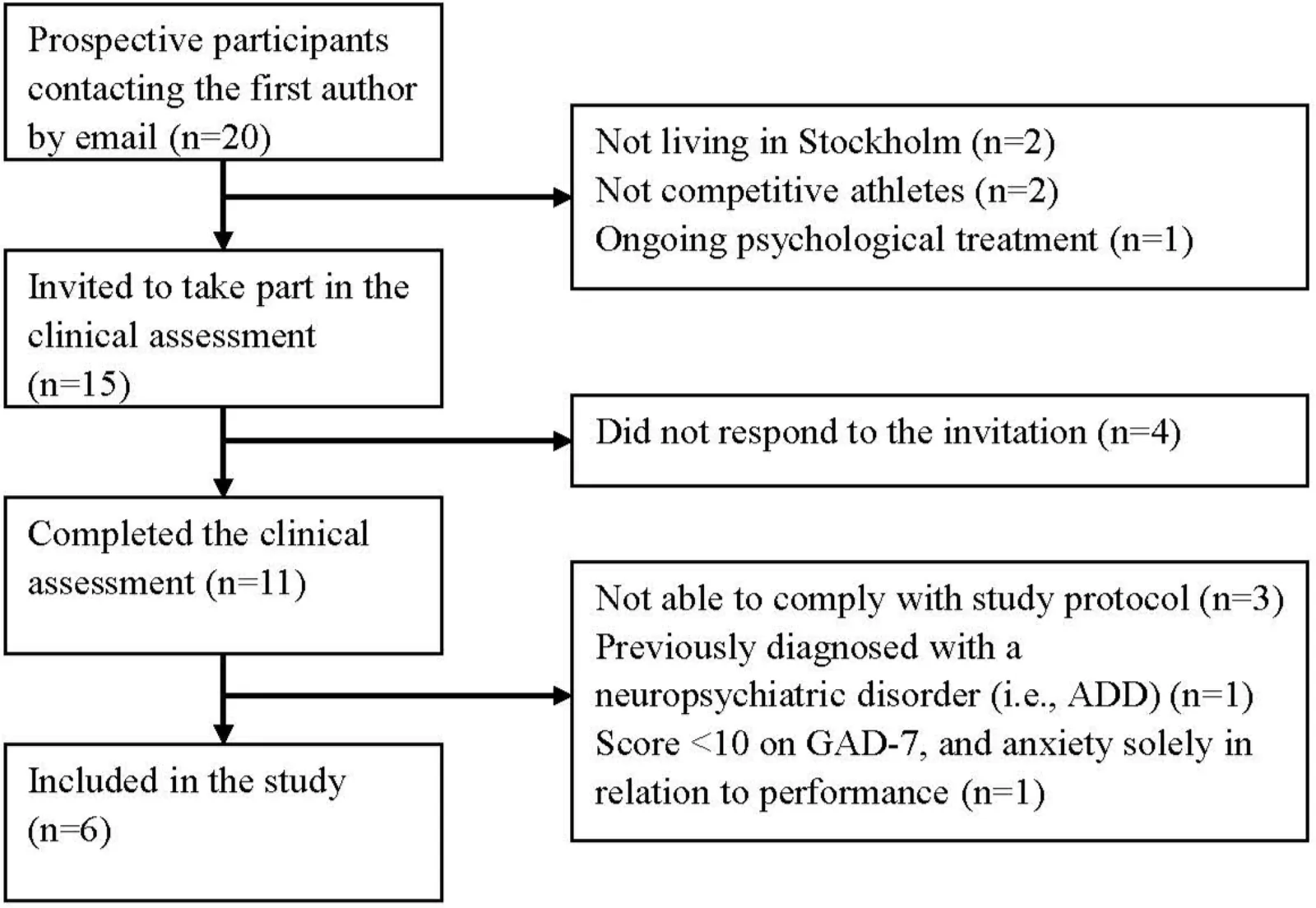
Participant recruitment flowchart

Participants that were excluded from the study after completing the clinical assessment were informed of this by the second author through phone calls and were recommended to seek treatment at their health care clinic. All participants that were offered to participate in the study accepted, and the first author invited each participant to an individual online EMA briefing session conducted through Microsoft Teams. The session lasted for 30 minutes and m-Path (i.e., a smartphone application and platform for real-time monitoring; Mestdagh et al., 2023) and the EMA questionnaire were discussed in detail. After the EMA briefing, the participants were contacted by the intervention facilitator to schedule the first session. The recruitment of participants and reasons for exclusion are presented in Figure 1.

Finally, the first author scheduled individual phone calls with each participant two days after baseline started, halfway through the intervention (i.e., after three sessions), and the day after the final session. These phone calls served as validity checks (see Schorrlepp et al., 2025) and were conducted to allow participants to ask questions, raise any concerns, and ensure adherence to the planned data collection procedure.

### Intervention

The intervention consisted of weekly CBT sessions on the same weekday every week for six weeks, each lasting 45 minutes. Treatment was followed as it naturally occurs in the clinic, without alteration or manipulation (i.e., passive intervention; Kazdin, 2021b). During the first session the psychologist conducted an individual conceptualization (Hofmann et al., 2013) and clarified an individual treatment goal. Based on the conceptualization, the content of the sessions was determined and individualized by the intervention facilitator who adapted the treatment to each participant’s needs and treatment goals. Details about each participant’s sessions are provided in Appendix A.

### Measures

#### Clinical assessment

Anxiety symptoms were assessed using the seven-item Generalised Anxiety Disorder 7-item scale (GAD-7; Spitzer et al., 2006). The GAD-7 includes seven items such as “Feeling nervous, anxious or on edge”, “Not being able to stop or control worrying”, and “Trouble relaxing”, rated on a 4-point Likert scale from 0 (*not at all*) to 3 (*nearly every day*). Total scores range from 0-21 with cut-off scores of 5, 10 and 15 indicating mild, moderate and severe anxiety, respectively. Scores of 10 or higher have been suggested to be useful for detecting other anxiety disorders such as social anxiety, panic disorder, and PTSD (Kroenke et al., 2007). The GAD-7 has demonstrated good internal consistency (α = 0.89, Löwe et al., 2008).

The Mini-International Neuropsychiatric Interview (M.I.N.I) Swedish version 7.0.0 (Sheehan et al., 1998) was used to screen for psychiatric disorders (e.g., MDD, panic disorder, agoraphobia, social phobia, OCD, GAD, anorexia nervosa, bulimia, PTSD).

#### Ecological momentary assessment

The premium version of m-Path (for details see https://mpath.io/manual/) was used to digitally collect daily life momentary assessments of positive affect, symptoms of anxiety, rumination, and acceptance. Time-sampling and a semi-random sampling scheme was employed. The feasibility of this approach in a clinical setting with athletes has been demonstrated in a prior study involving athletes undergoing CBT, supporting its application in the present context (Ekelund et al., 2025b).

Participants received four daily questionnaires at random time points in each of four 210-minutes time blocks between 8 a.m. and 10 p.m. Participants received a reminder notification 10 minutes after the original notification. Allowed response delay for a single questionnaire was set to 20 minutes, and allowed completion time of each item was 90 seconds. The daily EMA questionnaire consisted of 13 items during baseline and 14 items during the intervention phase. Of these, 13 items measured variables included in the current study, while one additional item was administered during the intervention phase only and was not included in the analyses.

The items measuring the outcome variables (i.e., symptoms of anxiety and positive affect) were responded to on a 7-point Likert scale from 1 (*not at all*) to 7 (*very*). Anxiety symptoms were measured with four items: “I feel worried”, “I feel unfocused”, “I feel annoyed”, and “I feel restless.” Positive affect was measured with three items: “I feel happy”, “I feel content”, and “I feel relaxed”.

The items measuring the mediating variables (i.e., rumination and acceptance) were prompted with a starting instruction “*Since the last beep*”. Rumination was measured with two items: “I have ruminated over things that have already happened” and “I have thought about the same thing over and over again” and were responded to a 7-point Likert scale from 1 (*rarely*) to 7 (*often*), where higher scores indicate higher levels of rumination.

Based on feedback from all participants two days after the baseline started, we had to revise the two items regarding acceptance in the questionnaire due to ambiguity, which posed a risk of response errors and misinterpretation. The revised items measured acceptance using a two-step item. The initial questions were: “I have had distressing thoughts” and “I have had distressing emotions”, to which participants responded “yes” or “no”. A “yes” response to either question triggered a corresponding follow-up: “When distressing thoughts have arisen, I have let them come and go without immediately trying to get rid of them” or “When distressing emotions have arisen, I have let them come and go without immediately trying to get rid of them.” The items were responded to on a 7-point Likert scale from 1 (*No, I have immediately tried to get rid of them*) to 4 (*Sometimes*) to 7 (*Yes, I have let them come and go*) where higher scores indicate higher levels of acceptance. Mean item scores were computed for all study variables included in the analyses (i.e., anxiety, positive affect, acceptance, and rumination)

### Analyses

#### Treatment effect

Visual analysis was preregistered as one approach to analyzing treatment effects as it is commonly used in single-case research (Ledford et al., 2017). However, due to the highly variable nature of the intensive longitudinal data, including variability in response rates and response patterns across participants, participants’ time series differed significantly between individuals. As a result, graphical visualization and visual analysis would be based on non-comparable data and risk being misleading when assessing change over time (Lanovaz & Turgeon, 2020; Jusko et al., 2025). Therefore, no visual analysis was conducted. This is a deviation from the preregistration and more detailed information regarding this change is available at https://osf.io/nwqrp/overview?view_only=668945f1e8514d03bec0410db984962a.

Tau was used to estimate the effect of the intervention and is one of the most frequently used quantitative analysis within single-case research (Fingerhut et al., 2020). Tau is a complete non-overlap effect size measure, meaning that it equally emphasizes all data points when measuring the between-phase change by examining the degree of overlap between phases (Kratochwill & Levin, 2014; Parker et al., 2011). Many variants of the Tau exist and hence recommendations from Fingerhut et al. (2021) were followed in selecting the proper Tau for the current study.

First, baseline trend was controlled for using the Baseline corrected tau calculator (Tarlow, 2016). Second, the effect size for all variables (i.e., anxiety, positive affect, rumination, acceptance) was calculated using the ShinyApp application for single-case effect size calculation (Pustejovsky et al., 2024). If a baseline trend was present, baseline corrected Tau (Tau-BC; Tarlow, 2017) was calculated while adjusting for trend using the Theil-Sen regression (Pustejovsky et al., 2024; Tarlow, 2017). If no baseline trend was present, no baseline adjustment was made and the effect size was calculated using the Tau (non-overlap) index (Pustejovsky et al., 2024). Confidence intervals were set to 95%. Effect sizes were interpreted as small (<0.20), moderate (0.20–0.60), large (0.60–0.80), and very large (>0.80; Vannest & Ninci, 2015).

In the single-case effect size calculator, direction of improvement for all study variables were specified (Pustejovksy et al., 2024). For anxiety and rumination, direction of improvement was specified as “all decrease” meaning that positive effect sizes are interpreted as a decrease in anxiety and rumination, and negative effect sizes are interpreted as an increase in anxiety and rumination. For positive affect and acceptance, direction of improvement was specified as “all increase” meaning that positive effect sizes are interpreted as an increase in positive affect and acceptance, and negative effect sizes are interpreted as a decrease in positive affect and acceptance.

#### Mediation analyses

The preregistration of the current study stated that the mediating effect of the intervention would be analyzed with single-case casual mediation analysis (sccm; Langenberg et al., 2022), using the R package *sccm* (Langenberg, 2021). However, the majority of the sccm-based analyses did not converge properly, and we therefore abandoned the sccm analysis and used a different approach to address the second research question (i.e., mediated effects) in the current study, which is described below.

**Figure 2:**
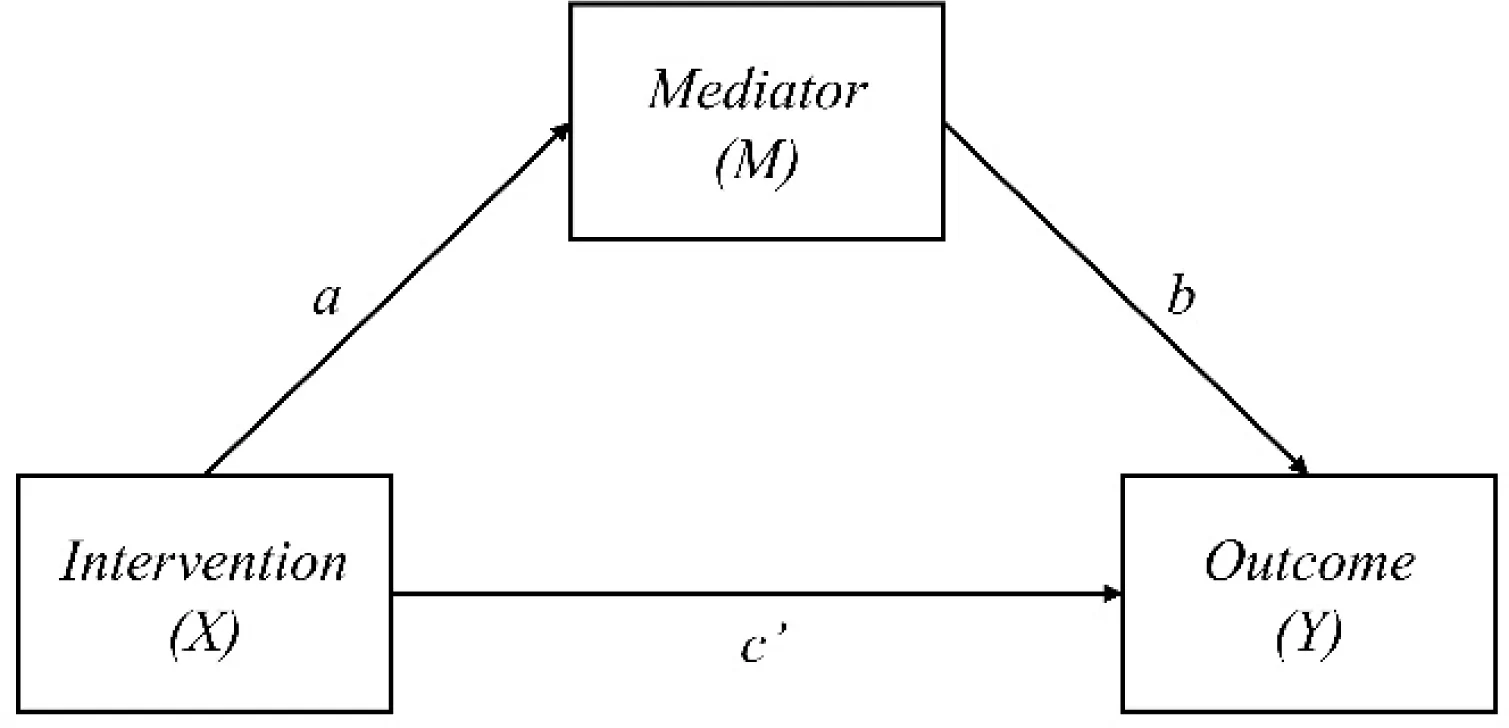
Single mediator model

Mediating effects of rumination and acceptance were analyzed in two steps using the Tau or Tau-BC and crosslagged correlations (cf. Geuke et al., 2019). Tau or Tau-BC was calculated to assess the effect of the intervention on rumination and acceptance (i.e., the *a*-path in a single mediator model. See Figure 2). The same procedure was employed for the Tau and Tau-BC calculations for rumination and acceptance as for the outcomes (i.e., anxiety and positive affect).

We used cross-lagged correlations to examine the change process during treatment and how changes in the mediators are associated with changes in the outcome variables (Gueke et al., 2019). The cross-lagged correlation between a mediator and an outcome can be used as a proxy for the *b* path in mediation analysis (Gueke et al., 2019) and the analysis provides information about the association between two time series, tested both forward and backward over time, after adjusting for autocorrelation (Borckardt et al., 2008). We used the Simulation Modeling Analysis software available at https://www.clinicalresearcher.org/software.htm to compute the cross-lagged correlations, and we estimated and compared lags at -5 to +5. If the significant lags are greater than zero (i.e., lag+1 to lag+5) it indicates that change in the mediator precedes change in the outcome, whereas significant lags that are negative (i.e., lag-1 to lag-5) indicates that change in the outcome precedes change in the mediator (i.e., indication of reversed causality). Statistically significant cross-lagged correlations at lag0 indicate that the mediator changes contemporaneously with the outcome. Data were aggregated across days prior to conducting the cross-lagged correlations and listwise deletion of missing data was used in the analysis (cf. Gueke et al., 2019). The significance level was set to 0.05 in the cross-lagged correlation analysis.

A statistically significant Tau or Tau-BC for the mediators in the expected direction (i.e., an increase in acceptance, and a decrease in rumination) combined with a cross-lagged correlation in the expected direction (i.e., an increase in acceptance precedes a decrease in anxiety and an increase in positive affect, and a decrease in rumination precedes a decrease in anxiety and an increase in positive affect) between the mediators and outcomes was interpreted as an indication of a mediated process.

## Results

Response rates ranged from 31% to 85%, with an average questionnaire response time of 46 seconds across all participants. Descriptive statistics (means and range for all study variables, and total number of days in the baseline phase and full study period) are presented in Table 2.

**Table 2:** Descriptives of study period, response rates, mean and range of each study variable

Participant	*N* days baseline	*N* total study days	Response rate %	Rumination		Acceptance		Anxiety		Positive affect	
				*M*	Range	*M*	Range	*M*	Range	*M*	Range
P1	5	48	31	A = 4.54 B = 3.82	A = 2–6 B = 1–7	A = 5 B = 3.38	A = 4–6 B = 1–7	A = 2.54 B = 2.38	A = 2–4 B = 1–4	A = 5.22 B = 4.89	A = 3.67–6 B = 2–7
P2	6	49	74	A = 4.53 B = 3.44	A = 3–6.5 B = 1–7	A = 2.33 B = 3.39	A = 1–3 B = 1–6	A = 2.88 B = 3.05	A = 1.5–4.25 B = 1.75–7	A = 4.27 B = 4.41	A = 3–6 B = 1.67–6.33
P3	12	55	50	A = 6.45 B = 4.02	A = 4.5–7 B = 1.5–7	A = 4.87 B = 6.02	A = 3–6 B = 4–7	A = 2.96 B = 2.21	A = 1.25–6.25 B = 1–4.5	A = 5 B = 5.62	A = 2.33–7 B = 3.33–7
P4	13	56	85	A = 3.72 B = 3.56	A = 2–6 B = 2–6	A = 2.56 B = 4.12	A = 2–4.5 B = 2–6	A = 3 B = 2.83	A = 1.5–5.5 B = 1.75–5	A = 3.57 B = 3.78	A = 1–5.67 B = 1.33–5.33
P5	19	63	56	A = 3.9 B = 3.09	A = 1–7 B = 1–7	A = 3.21 B = 3.95	A = 1–6 B = 1–7	A = 2.98 B = 2.40	A = 1.5–4.5 B = 1–4.75	A = 3.99 B = 4.50	A = 1.67–6 B = 2.33–6
P6	20	70	71	A = 2.98 B = 3.26	A = 1–6.5 B = 1–7	A = 4.83 B = 6.38	A = 3–7 B = 5.5–7	A = 3.45 B = 2.26	A = 1.25–5.75 B = 1–5	A = 3.55 B = 4.24	A = 1–6 B = 1.33–7

*Note*. A = baseline phase, B = intervention phase

### Effectiveness of the intervention on anxiety and positive affect

Results from the Tau and Tau-BC analyses of the intervention effect on anxiety and positive affect are presented in Table 3. Two participants (P1 and P4) did not show statistically significant effect sizes of the intervention for either anxiety or positive affect. P3 and P5 showed statistically significant and moderate effect sizes for both anxiety and positive affect, indicating lower levels of anxiety and higher levels of positive affect. P2 and P6 showed statistically significant large and moderate negative effect sizes, respectively, of the intervention on anxiety, indicating higher levels of anxiety. P2 showed a non-significant effect size on positive affect and P6 showed a moderate and statistically significant effect size on positive affect, indicating higher levels of positive affect.

**Table 3:** Results from Tau and Tau-BC analyses

Participant	Rumination			Acceptance			Anxiety			Positive affect		
	Est^b^	SE	95% CI [LL, UL]	Est^b^	SE	95% CI [LL, UL]	Est^b^	SE	95% CI [LL, UL]	Est^b^	SE	95% CI [LL, UL]
P1	.21	.15	[-.16, .51]	NA	NA	NA	.14	.17	[-.22, .46]	-.23	.17	[-.53, .13]
P2	-.99^a^*	.01	[-1, -.86]	.42	.27	[-.23, -.80]	-.99^a^*	.01	[-1, -.87]	.13	.16	[-.14, .37]
P3	.80*	.06	[.58, .91]	.65*	.09	[.35, .82]	.40*	.12	[.13, .61]	.26*	.14	[.02, .50]
P4	.07	.10	[-.13, .25]	.82*	.07	[.63, .92]	.08	.11	[-.12, .26]	.11	.11	[-.08, .29]
P5	.22*	.13	[.00, .42]	.21	.20	[-.17, .53]	.41*	.10	[.19, .58]	.29*	.11	[.07, .48]
P6	-.12	.09	[-.29, .05]	.85*	.07	[.66, .93]	-.40^a^*	.07	[-.55, -.24]	.35*	.09	[.18, .50]

*Note*.

b = for rumination and anxiety, positive effect sizes indicate a decrease and negative effect sizes indicate an increase.

For acceptance and positive affect, positive effect sizes indicate an increase and negative effect sizes indicate a decrease;

NA = results could not be estimated due to <3 data points in baseline;

a = adjusted for baseline trend;

* = statistical significance is indicated by 95% CIs that exclude zero

### Analyses of the Mediators

#### Tau and Tau-BC (a-path)

Results from the Tau and Tau-BC analyses on the effects of the intervention on the mediators (acceptance and rumination) are presented in Table 3.

**Acceptance**. For P1, the effect of the intervention on acceptance could not be estimated due to insufficient number of data points in baseline (<3) for Tau-BC analysis. For three participants (P3, P4, P6) a very large positive effect size on acceptance was observed, which provides support for the *a*-path of a mediated process. No statistically significant effect sizes were observed for P2 and P5.

**Rumination**. For three participants (P1, P4, P6) the effect sizes of the intervention on rumination was non-significant. Two participants (P3 and P5) showed moderate to large effect sizes on rumination indicating evidence for the *a*-path of a mediated process. For P2 a large negative effect size was observed indicating that the intervention increased levels of rumination, which is inconsistent with the expected direction of the mediated process.

#### Cross-lagged correlations (b-path)

**Figure 3:**
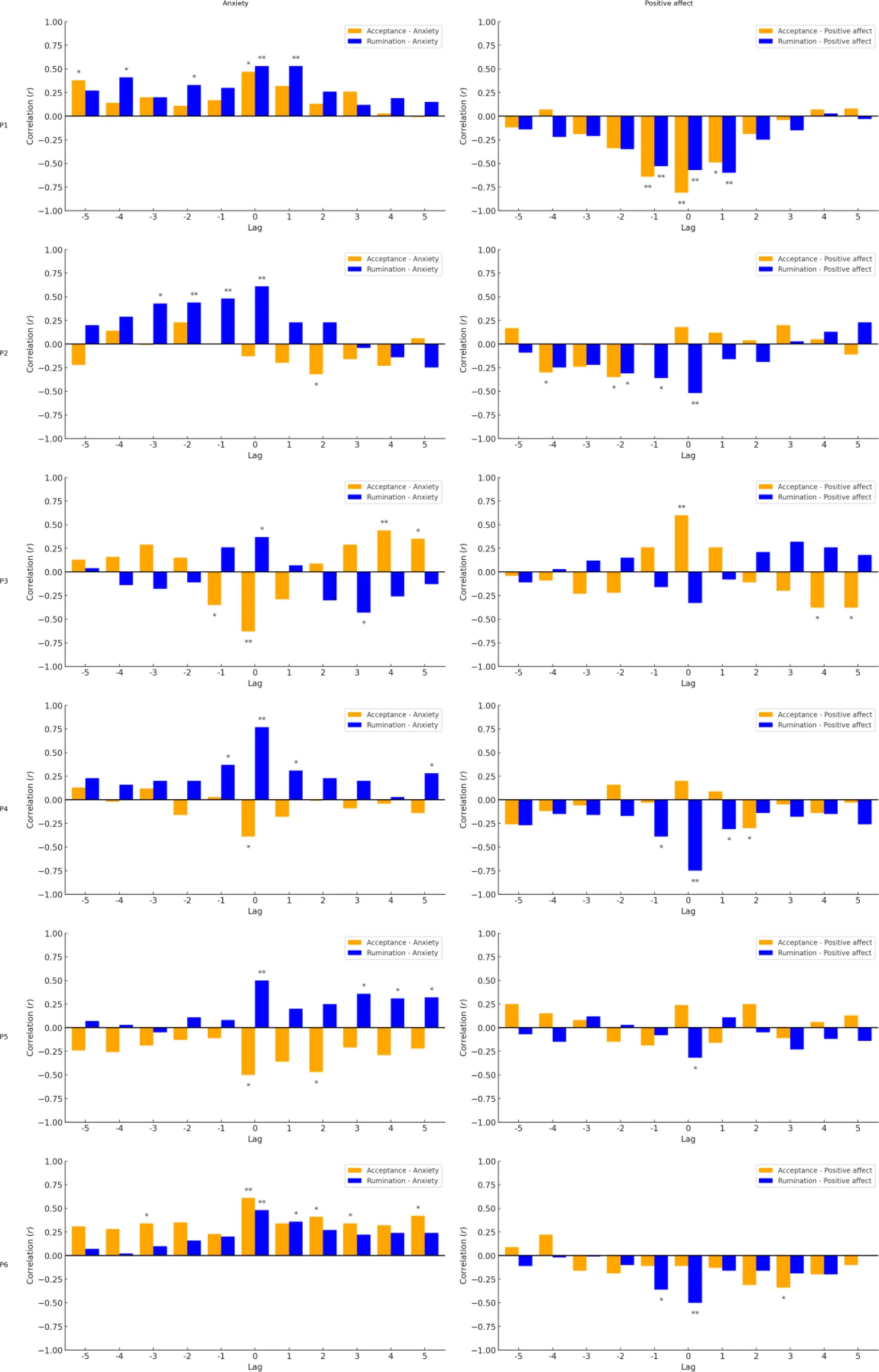
Cross-lagged correlations *Note*. Positive lags (lag +1 to +5) indicate that changes in the mediator precede changes in the outcome, whereas negative lags (lag −1 to −5) indicate that changes in the outcome precede changes in the mediator. Significant correlations at lag0 indicate contemporaneous change.

Results from the cross-lagged correlations between the mediators (acceptance and rumination) and the outcomes (anxiety symptoms and positive affect) are presented in Figure 3 and the specific correlations for each individual, variable, and lag can be found in Appendix B.

**Acceptance**. For anxiety, P1 showed a pattern of reversed causality as indicated by statistically significant negative lags whereas P2, P3, P5, and P6 showed some cross-lagged correlations indicating that changes in acceptance preceded changes in anxiety (i.e., significant positive lags). However, for P3, the direction of the cross-lagged correlations varied across lags, with a negative correlation at lag0 indicating contemporaneous change for acceptance and anxiety, and positive correlations at lag 4–5 indicating that an increase in acceptance was related to an increase in anxiety the following days (or vice versa, that a decrease in acceptance was related to a subsequent decrease in anxiety). The cross-lagged correlations for P4 indicated a contemporaneous change for acceptance and anxiety. For P6, although statistically significant cross-lagged correlations were observed at positive lags, the correlations were positive, indicating that increases in acceptance were followed by increases in anxiety (or vice versa, that a decrease in acceptance was related to a subsequent decrease in anxiety).

For positive affect, P1 showed the strongest cross-lagged correlations at lag0, indicating no temporal mediation but rather a contemporaneous change. Notably, higher acceptance was concurrently associated with lower positive affect for P1. The cross-lagged correlations for P2 were in line with a pattern of reversed causality, as indicated by significant negative lags for positive affect. For P3, the strongest cross-lagged correlations were also showed at lag0, indicating contemporaneous positive changes in acceptance and positive affect. P3, P4, and P6 showed some cross-lagged correlations, indicating that a change in acceptance preceded change in positive affect. However, for all three participants (P3, P4, P6) the correlations were negative, indicating that an increase in acceptance was related to a subsequent decrease in positive affect (or vice versa that a decrease in acceptance was related to a subsequent increase in positive affect). P5 did not show any statistically significant cross-lagged correlations between acceptance and positive affect.

**Rumination**. For anxiety, P1, P2, P4, P5, and P6 showed the strongest cross-lagged correlations at or near lag0, which indicates contemporaneous change. Furthermore, P1 and P2 showed a pattern of revered causality, whereas P3, P4, P5, and P6 showed some cross-lagged correlations in line with a mediated process through rumination on anxiety as indicated by significant positive lags. However, for P3 the direction of the cross-lagged correlations varied across lags, showing both positive and negative correlations.

For positive affect, P1, P4, P5, and P6 showed the strongest cross-lagged correlations at or near lag0. This pattern suggests primarily a contemporaneous change between rumination and positive affect rather than a temporal mediation process. For P2, a pattern of reversed causality was observed as indicated by significant negative lags, whereas for P3 no significant cross-lagged correlations were observed.

#### Results of the full mediation model

When integrating the results from the Tau and Tau-BC analyses with the cross-lagged correlations into the full conceptualized mediation models, none of the participants showed consistent evidence in line with a mediated process in the expected direction across both mediators (acceptance and rumination) and outcomes (anxiety and positive affect). Across all combinations of mediators and outcomes, only P5 met the criteria for a mediation process for rumination as a mediator of the effect on anxiety.

## Discussion

The first aim of the current study was to examine the effects of CBT on moderate to severe symptoms of anxiety and positive affect in athletes. The results demonstrate variability in treatment response, with some participants showing improvements while others showing no change or even an increase in anxiety symptoms. The second aim was to investigate the mediated effects of CBT on anxiety symptoms and positive affect through rumination and acceptance. Overall, evidence for acceptance as a mediator was heterogeneous across participants with somewhat unexpected results for some participants when higher acceptance was related to negative outcomes in terms of increased anxiety symptoms for two participants and less positive affect. Likewise, the support of rumination as a mediator of the effects of CBT on anxiety and positive affect was heterogenous across participants, with support in one participant (P5) for a mediated effect through rumination on anxiety, and no support for mediation through acceptance or rumination on positive affect.

The reality of real-world clinical practice becomes visible in the findings of the current study. Despite examining the results in light of individual baseline clinical characteristics and the specific intervention content for each participant, it is challenging to identify factors that explain why CBT was effective for some but not for others. The participants showed both shared clinical characteristics and individual differences but notably is that all participants had a history of MDD or an ongoing MDD which highlights the comorbidity of MDD and symptoms of anxiety among the participants. This clinical representation aligns with previous research showing the occurrence of comorbidity in athlete populations (Oevreboe et al., 2023). Specifically in a Swedish population of elite athletes, 22% of female elite athletes who were seeking treatment at a clinic specializing in sport psychology had comorbid anxiety and an affective disorder (e.g., MDD; Åkesdotter et al., 2022).

Moreover, three participants in the current study also had comorbid anxiety disorders. A review and meta-analysis on the determinants of anxiety in elite athletes identified comorbid diagnoses, including GAD with depression, as well as co-occurrences of multiple anxiety disorders, such as GAD with OCD, agoraphobia, or panic disorder (Rice et al., 2019). The heterogenous results of the current study in combination with previous descriptive findings about anxiety disorders in the athlete population not only highlights the complexity of mental health problems, but also the importance of recognizing individual variability in treatment outcomes in this population when providing recommendations for treatment.

An interesting finding of the current study is that increased acceptance was related to negative outcomes (i.e., higher levels of anxiety symptoms and lower levels of positive affect) in the short term (one day to the next) for three participants. This finding highlights the potential short term emotional costs of actively engaging with distressing thoughts and emotions, which is central to acceptance-based strategies that emphasize becoming aware of one’s emotional experiences rather than attempting to avoid or control them (Hayes & Hofmann, 2021).

However, the literature on acceptance as an emotion regulation strategy and its effect on negative affect is inconsistent (Kohl et al., 2012). One possible explanation for the mixed findings is the difficulty of measuring a complex process such as acceptance (Stockton et al., 2019). In laboratory settings, acceptance has been shown both to increase negative affect (Troy et al., 2017) and to reduce negative affect when exposed to stressors (Ford et al., 2018).

Moreover, Ford et al. (2018) conclude that the use of acceptance does not involve “collateral damage” in terms of reduced positive affect, and contemporaneous emotional regulation at the daily level have shown that acceptance was significantly and positively related to positive affect (Boemo et al., 2022). This is contradictory of the results of the current study for two participants, for whom higher levels of acceptance were associated with lower levels of positive affect. Such findings underscore the individual variability in the potential short-term emotional costs associated with using emotional regulation strategies such as acceptance.

While some evidence suggests that acceptance may be more effective in clinical samples (Kohl et al., 2012), other findings indicate that acceptance may not be appropriate for individuals with clinical mental health problems such as anxiety and depression (Troy et al., 2017). These previous findings combined with the findings of the current study suggest that it remains unclear how acceptance influences subjective emotional experiences in the short term in real-world clinical settings during psychotherapy.

Furthermore, evidence of mediation was only observed in part for one participant, suggesting limited support for the mediation models and substantial heterogeneity across participants. The limited support for the mediation models relates to the broader question of how psychological change is conceptualized and how it unfolds over time (Hayes et al., 2007). Any change during psychotherapy is often assumed to be linear and occur in a stepwise manner, where shifts in one process precede and casually influence changes in another (Hayes et al., 2007). This also relates to the criteria of temporal precedence for establishing mediation, where a change in the mediator is observed before a change in the outcome (MacKinnon, 2011). However, the present findings suggest that changes in anxiety or positive affect and changes in rumination and acceptance primarily occurred concurrently rather than sequentially. This pattern indicates that these processes may influence each other within the same time frame (i.e., day), rather than across successive time points (i.e., days) as implied by a stepwise mediation model.

These findings are consistent with the findings of a meta-analysis of mediation in acceptance- and mindfulness-based interventions, which reported that studies using correlation-based mediation analyses were more likely to demonstrate statistically significant mediating effects, whereas studies employing time-lagged designs did not yield significant mediating effects (Johannsen et al., 2022). Together, these findings highlight remaining gaps in our understanding of how psychological change unfolds over time, as well as the extent to which conclusions depend on the design and analytic approach used (Hamaker, 2025). More fine-grained analyses conducted within shorter time frames (e.g., within days) and across longer observational periods may deepen our understanding of how psychological change processes unfold in clinical samples and athletes during treatment.

### Limitations

Despite its contributions, the current study is not without limitations. First, no manipulation of the independent variable (i.e., the intervention) was made. In an intervention study with psychotherapy one common manipulation of the intervention is to provide a standardized treatment protocol for the intervention facilitator and the participants to follow (Hofmann et al., 2012). That said, the use of treatment protocols assumes that all individuals will respond the same to the same intervention components which violates the essence of a person-centered focus and single-case approaches (Hayes et al., 2022; Hayes & Hofmann, 2021; Kazdin, 2021a). Nevertheless, no manipulation of the intervention could be considered a threat to internal validity (Tate et al., 2013). However, the current study limits threats to internal validity by implementing a concurrent baseline and randomization to intervention start, which are two of the main approaches for eliminating threats to internal validity in SCEDs (Tate et al., 2013).

Second, the results should be interpreted in light of methodological limitations related to capturing complex psychological processes in real-time. In the current study, all items were designed by the study authors and were guided by current methodological efforts to standardize (Eisele et al., 2025), openly share (Kirtley et al., 2020), and validate EMA items (e.g., Stinson et al., 2022; Schorrlepp et al., 2025). Even so, the two items measuring acceptance had to be revised after two days of baseline after participants reported difficulties interpreting them which posed a threat to item validity. This situation highlights the importance of incorporating opportunities in the study design to collect qualitative information (e.g., personal phone calls) throughout the study to allow for validity checks and to enable adjustments that may improve data collection and ensure item validity when using EMA (Schorrlepp et al., 2025).

Furthermore, since the validity of the acceptance items during the initial baseline phase could not be ensured, measurements of acceptance from the first two days of baseline were removed from all analyses. Despite efforts to improve the acceptance items, the acceptance variable proved difficult to include in the analyses. The results related to this construct should be interpreted with caution due to the reduced number of baseline observations and the higher proportion of missing data for this variable compared to the other variables.

Third, the study aimed to investigate the mediating effects of CBT on anxiety symptoms and positive affect through rumination and acceptance. Due to difficulties related to the acceptance variable, the preregistered mediation analysis (i.e., sccm; Langenberg et al., 2022) could not be conducted as planned, and indirect effects were therefore not estimated directly. Instead, mediation was examined by separating the *a*- and *b*-paths of a single mediation model into two distinct analyses (i.e., Tau or Tau-BC and cross-lagged correlations), which constitutes a methodological limitation of the mediation analysis.

This limitation is, however, not unique to the current study but reflects broader methodological challenges with mediation analysis within single-case research. A recent systematic review examining mediator characteristics in single-case designs found that no studies reported effect sizes for indirect mediation effects (Moeyaert et al., 2025). This suggests that, despite ongoing methodological efforts to advance mediation analysis in single-case research (e.g., Langenberg et al., 2022; MacKinnon et al., 2022; Miočević et al., 2022), considerable challenges remain when applying these approaches to real-world clinical data.

### Future directions

The individual variability observed in the current study demonstrate the need to complement existing group-based research with high-quality SCEDs and intensive longitudinal data. Both single-case research and high-density data collection (e.g., EMA) is gaining momentum (Fritz et al., 2024; Vlaeyen et al., 2020), and increasing emphasis is being placed on the individual (Hayes et al., 2022; Moskow et al., 2023), along with accompanying methodological recommendations for individual-level analyses (Hamaker, 2025). Continuing and building on this line of research would allow a better understanding of intervention effects at the individual level, as well as the change processes and mechanisms that underlie therapeutic outcomes.

Furthermore, researchers conducting SCEDs in combination with high-density data collections should consider including qualitative measures to accompany the quantitative analyses to deepen the understanding of the individual trajectories over time (Olthof et al., 2024; Schorrlepp et al., 2025). Specifically for single-case research, social validation can be used to contextualize the findings and account for clinical significance (Kazdin, 2021b). However, this is often done after the intervention is completed, while qualitative EMA measures are collected throughout the intervention. These types of qualitative measures can be especially valuable in terms of understanding negative treatment effects (Rosendahl et al., 2025), but also to understand what makes treatment successful. For elite athletes specifically, qualitative measures can help with information about specific sport contextual factors (e.g., competition schedule, injuries, setbacks) which in turn can deepen the understanding of the individual trajectories during an intervention.

Finally, future research in clinical sport psychology should aim to include both male and female athletes. Although female athletes have been found to have a higher occurrence of developing eating disorders, depression, and anxiety compared to male athletes (Rungstrøm et al., 2024), male athletes remain underrepresented in clinical treatment studies with athletes (Ekelund et al., 2025a). Including male athletes would contribute to a more comprehensive understanding of treatment effects and individual variability in clinical athlete populations.

## Conclusion

This study is the first to examine the effects of cognitive behavioral therapy using a SCED combined with EMA in competitive athletes with subclinical and clinical symptoms of anxiety. The study demonstrates substantial individual variability in treatment effect and mediating processes during cognitive behavioral therapy. This variability underscores the importance of continued investigation of individual treatment effects and underlying processes through idio-graphic and ecologically valid designs. Accumulating evidence from such idiographic research may help clinicians to tailor interventions more precisely and support researchers within both clinical and sport psychology in the development of therapies that benefit not only athletes but also the general population.

## Data Availability

Data will be made available on request.

## Action editor

Lars-Gunnar Lundh served as action editor for this article.

## Open access

This article is distributed under the terms of the Creative Commons Attribution 4.0 International License (http://creativecommons.org/licenses/by/4.0/), which permits unrestricted use, distribution, and reproduction in any medium, provided you give appropriate credit to the original author(s) and the source, provide a link to the Creative Commons license, and indicate if changes were made.

## Appendix A: Content of each of the sessions for all participants

**Table:**

ID	Session 1	Session 2	Session 3	Session 4	Session 5	Session 6
**1**	Psychological anamnesis Problem statement Goal formulation Psychoeducation **HA:** “Emotion journal”	Psychoeducation on affect Identify emotions (being present) Treat emotions neutrally (acceptance) **HA:** “Emotion wheel”. Behavioral experiment/exposure (linked to valuebased living)	Functional analysis Explorative about participant’s personality and how it has been shaped by its environment and surroundings while growing up, and how this relates to current behavioral patterns 1. Note thought/emotion (beingpresent) 2. Identify thought/emotion 3. Observe without judgment (acceptance, defusion) 4. Act with awareness towards valuebased living (value-based living, committed action) **HA:** Behavioral experiment with a focus on a specific functional behavior	Exploring how the participant can map contextual, personal, and physical conditions before a performance, to adjust the expectations, and later make a more fair evaluation of the actual performance, with the aim of reducing frustration when the participant does not succeed or perform as desired (also assigned as home assignment) Engage in mentalization regarding other people’s differences and reaction patterns, emphasizing the importance of acceptance when facing difficult situations. This helps prevent getting caught up in personal judgements about what is difficult to understand, unusal or “bad” (promotion of non-judgmental stand to increase psychological flexibility) Conversation about selfimage, selfidentity **HA:** Mapping value-based living Keep practicing on affect awareness and functional emotion regulation	Psychoeducation on value-based living Mapping of committed action Work with emotion regulation techniques (breathing in a square, body scanning for early detection of physiological emotion markers) **HA:** Evaluation of treatment goal	Evaluation of treatment goal Crisis management in response to a recent difficult event experienced by the participant, including psychoeducation about crisis situations and the importance of basic needs Normalization and validation of difficult thoughts and emotions
**2**	Psychological anamnesis Problem statement Goal formulation **HA:** Mapping of worry thoughts	Cognitive scheme Psychoeducation Cognitive restructuring **HA:** Being present and defusion (paus, try not to follow the thoughts)	1. Note thought/emotion (beingpresent) 2. Identify thought/emotion 3. Observe without judgment (acceptance, defusion) 4. Act with awareness towards valuebased living (value-based living, committed action) Defusion (“Now I have the thought that…”, “A thought is just a thought”, “A feeling is just a feeling”) Being present (stop and notice thought flow/emotions in the body) Self-as-context (metaphor: inner theatre, director vs actor) Thought traps (psychoeducation on jumping to conclusions when catastrophic thoughts pop up) **HA:** “Emotion wheel”	Psychoeducation on personality (Big 5) “Affect school” and introduction to primary and secondary affect Mapping of emotions and emotion patterns Conversation about the importance to live authentically and in line with one self (personality) instead of trying to be someone else because of pressure from the surrounding environment Conversation about preventive stress reduction strategies by prioritizing in every day life and scheduling of recovery **HA:** Body scanning Stress reduction strategies Value based living through committed action	Behavior analysis Emotional awareness Cognitive restructuring Conversation about how family dynamics in childhood has shaped current relational patterns and self-esteem **HA:** Behavioral experiment/exposure in line with committed action	Exploring of thought traps combined with cognitive restructuring related to ageing and phases of life Repetition of value-based living and committed action Evaluation of the treatment Mapping of risk factors ahead and exploring of strategies to prevent/cope with these
**3**	Psychological anamnesis Problem statement Goal formulation **HA:** Mapping value-based living	Review of home assignment Exposure Conversation about feeling compassion for and gratitude toward oneself 1. Note thought/emotion (being present) 2. Identify thought/emotion 3. Observe without judgment (acceptance, defusion) 4. Acting with awareness towards value-based living (value-based living, committed action) **HA:** Practice the method of four steps described above, and keep engaging in exposure to anxiety-related behaviors	Review of the home assignment Functional analysis to demonstrate the effect of a behavior on short and long term Mapping patterns of worry thoughts related to the reason for seeking treatment Explore how focus can be shifted from the mind to the body and practice to evaluate current psychological status through emotions rather than the thought **HA:** Exposure in everyday life. Identify negatively loaded words that relates to the problem statement	Reviewing the home assignment Mapping underscoring factors to increased stress in daily life Conversation about inner conflict regarding goals in daily life (sports vs private life), nuancing for increased psychological flexibility, and prioritizing of committed action **HA:** Continued engaging in exposure, and strategies for emotion regulation (defusion)	Review of the home assignment Psychoeducation of stress preventive strategies such as method for prioritizing in everyday life (have to do, should do, could do, want to do) Mapping of recovery activities and creating a plan for increased implementation of these No home assignment	Cognitive restructuring regarding a specific work related event experienced by the participant in the past week Exploring value-based living and committed action related to a specific challenge in one of the participant’s professional relations Evaluation of treatment goal
**4**	Psychological anamnesis Problem statement Goal formulation **HA:** “Worry diary” as well as identify the three most common anxiety-related behaviors	Mapping of anxietyrelated and compulsive behaviors Psychoeducation on dysfunctional and functional behaviors (frequency, duration, intensity) Anxiety curve Anxiety hierarchy Discuss mechanisms behind exposure **HA:** Exposure of anxiety-related behaviors	Functional analysis based on exposed anxiety-related behaviors from the last week Investigate how relational behavior patterns become dysfunctional in the long term Conversation about self-esteem and how self-esteem can be strengthened by expressing one’s own needs/wishes/wants/thoughts/emotions Brief psychoeducation on primary and secondary affect **HA:** Exposure of anxiety-related behaviors	“Affect school” and psychoeducation focused on anger, fear, worry, anxiety, and grief Primary and secondary affect Exploring of how one’s relationship with one’s own emotions was shaped in childhood by primary attachment figures “Emotion wheel” **HA:** Practice being present to enhance emotional awareness	Psychoeducation on stress prevention- and coping strategies such as prioritizing (have to do, should do, could do, want to do) Scheduling of recovery activities in everyday life Exploring of an episode of mental illness in the past and how this affects current thoughts and emotions. In relation to this - an exercise on staying in the moment and difficult emotions was conducted (being present and acceptance) **HA:** Schedule rest and work with prioritizing of tasks, as well as continue with exposure of anxiety-related behaviors	Repetition of previously run thought strategies Walk through of a strategy to move the focus on thoughts from the present to here and now Evaluation of treatment goal Exploring potential fall pits moving forward and signals to be aware of
**5**	Psychological anamnesis Problem statement Socratic questioning focused on underlying contributors to anxiety in previous life history **HA:** Mapping the most common worry thoughts	Review of the home assignment Mapping and nuancing self-image Conversation on the importance of embracing all aspects of oneself to foster selfcompassion and support the development of close relationships. In the long run, this also aims to reduce the tendency to project self-related values onto others in social situations, thereby reducing anxiety in accordance with the treatment goals **HA:** Mapping selfimage	Review of the home assignment Discussion on the importance of having non-judgmental and accepting approach to all aspects of oneself Psychoeducation on personality (big 5), and affects (mainly fear, worry, anxiety, anger and grief) Mapping of primary and secondary affects Functional analysis based on current dysfunctional emotional regulation strategies Exploring relational experiences and care patterns from childhood, and how these have affected self-esteem and one’s approach to emotional needs **HA:** Staying in the primary affect - exposure to difficult emotions	Review of home assignment Mapping of relations that trigger difficult emotions, as well as finding common patterns with these relationships Discussion on the importance of boundary setting in a selfrespective purpose, as well as explore what committed actions that align with this **HA:** Continue working with being present and acceptance of difficult emotions, as well as actively seek functional relationships where boundaries are respected	Functional analysis based on behavior experiment to live more authentically (being present, acceptance, valuebased living, committed action) Exploring the balance between allowing oneself to think and feel, but being selective about what thoughts and emotions that are functional/helpful to act on Exploration of the participant’s stance toward their own traumas and how this has been shaped by significant others’ way of relating both to themselves and to the participant Validating and helping to facilitate acceptance of difficult emotions Brief psychoeducation about the effective mechanisms of trauma treatment **HA:** Exposure to living and acting in accordance with value-based living	Review of the home assignment Behavior analysis based on experienced challenges in a current close relationship, and mapping of maintenance behaviors Conclusion and evaluation of the treatment
**6**	Psychological anamnesis Problem statement Goal formulation	Functional analysis Thought traps 1. Note thought/emotion (being present) 2. Identifythought/emotion 3. Observe withoutjudgment(acceptance,defusion) 4. Act withawareness and inline with value-based living (value-based living,committed action) Defusion (“I have a thought”, “A thought is just a thought”, “A feeling is just a feeling”) **HA:** Acting with awareness in line with value-based living	Expolartive talk about the function of the participant’s rumination and worry, combined with cognitive restructuring and creating awareness about thought traps* Mapping of dysfunctional avoidance behaviors that turn into self-fulfilling prophecies Introduction to value-based living and mapping of committed actions **HA:** Keep exploring value-based living and work with committed action as a complement to defusion when worrying thoughts appear **The participant repeatedly expressed that the third-wave strategies were difficult to understand, and difficult to apply in their everyday life. The psychologist therefore made the decision to include more traditional cognitive strategies such as cognitive restructuring to see if these better fitted the client and their needs*.	Creating alternative thoughts to current worry thoughts Body scanning (being present) **HA:** Use body scanning, finding alternative thoughts, defusion, and switching negatively loaded words to more neutral words	Functional analysis about exposure behaviors since previous session Mapping of dysfunctional stress coping behaviors and exploration of alternative functional behaviors Strategies for being present Defusion applied to “what-if-thoughts” **HA:** Trying functional behaviors for stress coping behaviors	Review of the home assignment Go through the prioritization tool (have to do, should do, could do, want to do) as a stress preventive strategy to continue working with Conclusion and evaluation of the treatment

*Note:* HA = home assignment

## Appendix B: Cross-Lagged Correlations Participant 1-3 (P1-P3)

**Table:**

Lag	*M* = Acceptance *Y* = Anxiety		*M* = Rumination *Y* = Anxiety		*M* = Acceptance *Y* = Positive affect		*M* = Rumination *Y* = Positive affect	
	*r*	*p*	*r*	*p*	*r*	*p*	*r*	*p*
P1								
-5	0.38	0.03	0.27	0.08	-0.12	0.31	-0.14	0.27
-4	0.14	0.26	0.41	0.02	0.07	0.40	-0.22	0.16
-3	0.20	0.18	0.20	0.17	-0.19	0.24	-0.21	0.18
-2	0.11	0.31	0.33	0.05	-0.34	0.11	-0.35	0.07
-1	0.17	0.23	0.30	0.07	-0.64	0.01	-0.53	0.01
0	0.47	0.02	0.53	0.00	-0.81	0.00	-0.57	0.00
+1	0.32	0.09	0.53	0.00	-0.49	0.04	-0.60	0.00
+2	0.13	0.28	0.26	0.11	-0.19	0.25	-0.25	0.14
+3	0.26	0.10	0.12	0.30	-0.04	0.44	-0.15	0.26
+4	0.03	0.44	0.19	0.17	0.07	0.39	0.03	0.45
+5	-0.01	0.48	0.15	0.23	0.08	0.37	-0.03	0.43
*n*	20		28		20		28	
P2								
-5	-0.22	0.09	0.20	0.15	0.17	0.16	-0.09	0.30
-4	0.14	0.21	0.29	0.06	-0.30	0.04	-0.25	0.08
-3	-0.01	0.48	0.43	0.01	-0.24	0.08	-0.22	0.11
-2	0.23	0.10	0.44	0.01	-0.35	0.03	-0.31	0.04
-1	0.00	0.51	0.48	0.00	-0.01	0.47	-0.36	0.03
0	-0.13	0.24	0.61	0.00	0.18	0.16	-0.52	0.00
+1	-0.20	0.14	0.23	0.11	0.12	0.27	-0.16	0.17
+2	-0.32	0.04	0.23	0.12	0.04	0.42	-0.19	0.14
+3	-0.16	0.19	-0.04	0.43	0.20	0.14	0.03	0.44
+4	-0.23	0.10	-0.14	0.23	0.05	0.39	0.13	0.23
+5	0.06	0.36	-0.25	0.08	-0.11	0.25	0.23	0.09
*n*	31		43		31		43	
P3								
-5	0.13	0.25	0.04	0.42	-0.04	0.42	-0.11	0.29
-4	0.16	0.21	-0.14	0.27	-0.09	0.31	0.03	0.46
-3	0.29	0.08	-0.18	0.21	-0.23	0.12	0.12	0.30
-2	0.15	0.24	-0.11	0.32	-0.22	0.14	0.15	0.25
-1	-0.35	0.05	0.26	0.11	0.26	0.10	-0.16	0.23
0	-0.63	0.00	0.37	0.05	0.60	0.00	-0.33	0.06
+1	-0.29	0.08	0.07	0.39	0.26	0.10	-0.08	0.36
+2	0.09	0.34	-0.30	0.09	-0.11	0.32	0.21	0.17
+3	0.29	0.08	-0.43	0.02	-0.20	0.17	0.32	0.06
+4	0.44	0.01	-0.26	0.10	-0.38	0.03	0.26	0.10
+5	0.35	0.03	-0.13	0.27	-0.38	0.02	0.18	0.18
*n*	37		38		37		38	
P4								
-5	0.13	0.22	0.23	0.08	-0.26	0.07	-0.27	0.05
-4	-0.02	0.45	0.16	0.16	-0.12	0.26	-0.15	0.20
-3	0.12	0.24	0.20	0.11	-0.06	0.37	-0.16	0.18
-2	-0.16	0.17	0.20	0.12	0.16	0.18	-0.17	0.17
-1	0.03	0.43	0.37	0.01	-0.03	0.45	-0.39	0.02
0	-0.39	0.01	0.77	0.00	0.20	0.15	-0.75	0.00
+1	-0.18	0.16	0.31	0.03	0.09	0.30	-0.31	0.04
+2	-0.01	0.49	0.23	0.08	-0.30	0.05	-0.14	0.22
+3	-0.09	0.31	0.20	0.11	-0.05	0.39	-0.18	0.15
+4	-0.04	0.41	0.03	0.42	-0.14	0.21	-0.15	0.19
+5	-0.14	0.20	0.28	0.03	-0.03	0.43	-0.26	0.07
*n*	32		43		32		43	
P5								
-5	-0.24	0.13	0.07	0.32	0.25	0.07	-0.07	0.32
-4	-0.26	0.11	0.03	0.43	0.15	0.21	-0.15	0.16
-3	-0.19	0.20	-0.05	0.39	0.08	0.33	0.12	0.21
-2	-0.13	0.28	0.11	0.24	-0.15	0.22	0.03	0.41
-1	-0.11	0.32	0.08	0.32	-0.19	0.16	-0.08	0.30
0	-0.50	0.02	0.50	0.00	0.24	0.11	-0.32	0.02
+1	-0.36	0.06	0.20	0.12	-0.16	0.21	0.11	0.26
+2	-0.47	0.02	0.25	0.06	0.25	0.09	-0.05	0.37
+3	-0.21	0.17	0.36	0.01	-0.11	0.27	-0.23	0.06
+4	-0.29	0.09	0.31	0.02	0.06	0.38	-0.12	0.20
+5	-0.22	0.16	0.32	0.02	0.13	0.22	-0.14	0.18
*n*	25		43		25		43	
P6								
-5	0.31	0.06	0.07	0.34	0.09	0.31	-0.11	0.22
-4	0.28	0.08	0.02	0.44	0.22	0.13	-0.02	0.47
-3	0.34	0.05	0.10	0.27	-0.16	0.21	-0.01	0.48
-2	0.35	0.06	0.16	0.17	-0.19	0.18	-0.10	0.28
-1	0.23	0.16	0.20	0.13	-0.11	0.30	-0.36	0.01
0	0.61	0.00	0.48	0.00	-0.11	0.31	-0.50	0.00
+1	0.34	0.06	0.36	0.02	-0.13	0.26	-0.16	0.16
+2	0.41	0.02	0.27	0.06	-0.31	0.07	-0.16	0.15
+3	0.34	0.04	0.22	0.10	-0.34	0.04	-0.19	0.12
+4	0.32	0.06	0.24	0.08	-0.20	0.15	-0.20	0.10
+5	0.42	0.01	0.24	0.07	-0.10	0.29	0.00	0.48
*n*	27		49		27		49	

*Note*. *M* = mediator, *Y* = outcome, *r* = correlation coefficient, *p* = *p*-value
